# A 3D numerical study of the collateral capacity of the circle of Willis with anatomical variation in the posterior circulation

**DOI:** 10.1186/1475-925X-14-S1-S11

**Published:** 2015-01-09

**Authors:** Yuan Ren, Qiang Chen, Zhi-Yong Li

**Affiliations:** 1 Biomechanics Laboratory, School of Biological Science & Medical Engineering, Southeast University, Nanjing 210096, China; 2 School of Chemistry, Physics and Mechanical Engineering, Queensland University of Technology, Brisbane, QLD 4001, Australia

## Abstract

**Background:**

The Circle of Willis (CoW) is the most important collateral pathway of the cerebral artery. The present study aims to investigate the collateral capacity of CoW with anatomical variation when unilateral internalcarotid artery (ICA) is occluded.

**Methods:**

Basing on MRI data, we have reconstructed eight 3D models with variations in the posterior circulation of the CoW and set four different degrees of stenosis in the right ICA, namely 24%, 43%, 64% and 79%, respectively. Finally, a total of 40 models are performed with computational fluid dynamics simulations. All of the simulations share the same boundary condition with static pressure and the volume flow rate (VFR) are obtained to evaluate their collateral capacity.

**Results:**

As for the middle cerebral artery (MCA) and the anterior cerebral artery (ACA), the transitional-type model possesses the best collateral capacity. But for the posterior cerebral artery (PCA), unilateral stenosis of ICA has the weakest influence on the unilateral posterior communicating artery (PCoA) absent model. We also find that the full fetal-type posterior circle of Willis is an utmost dangerous variation which must be paid more attention.

**Conclusion:**

The results demonstrate that different models have different collateral capacities in coping stenosis of unilateral ICA and these differences can be reflected by different outlets. The study could be used as a reference for neurosurgeon in choosing the best treatment strategy.

## Introduction

Stroke is a major health problem and a leading cause of adult disability in the world, and it ranks the second in the list of causes of death [1-4]. Wherein, atherosclerosis accounts for up to one-third of strokes [5]. Atherosclerosis of supra-aortic vessels and especially at the common carotid bifurcation is a major cause of recurrent ischaemic stroke [6]. There are two primary reasons for stroke caused by atherosclerosis: one is that the atheromatous plaque may be the source of cerebral emboli, which may result in cerebral infarction [7]. The other is that atherosclerosis leads to vascular stenosis and the decrease of vascular lumen, which may cause inadequate blood perfusion of the downstream cerebrovascular and brain tissue [8].

Carotid occlusive disease amenable to re-vascularization accounts for 5-12% of new strokes [9-11]. As for patients with severe internal carotid artery (ICA) stenosis, carotid endarterectomy (CEA) has been recognized as an effective therapy for re-vascularization [12,13]. However, in fact, it is often compared with carotid artery stenting (CAS), another therapy employed, when selecting the best treatment strategy for an individual patient. Thus, as an important index, the collateral capacity of the cerebral artery is very necessary to be pre-known [14].

The circle of Willis (CoW) is a ring-like arterial structure, and it links the two main cerebral artery systems, namely the internal carotid artery system and the vertebrobasilar system, and it is also the primary collateral pathway locating in the base of brain. When the blood flow of an unilateral artery declines caused by stenosis, it can be compensated from the contralateral side by the CoW. The collateral capacity of the CoW improves cerebral perfusion in ischemic areas and may diminish the effect of ischemic events [15,16]. Unfortunately, based on the radiological and anatomical studies [17,18], no more than 50% of the general population have a complete CoW. The possible variations include hypoplasia or completely absent blood vessels, which may occur in the anterior circulation or the posterior circulation. The variations may influence the collateral capacity of the CoW and the risk of ischaemic stroke. Therefore, evaluating the collateral capacity of different configurable CoWs in patients is very important [19].

There have been some studies performed on haemodynamics of CoW with different anatomical variations in recent years. Some researchers have treated the cerebral vasculature as 1D structure, however, basing on Poiseuille flow, it cannot capture the effects of the complex arterial geometry, especially the effects of blood vessel junctions [20,21]. Although 2D models can improve the accuracy of simulation [22], it's still necessary to establish 3D models for obtaining the more realistic hemodynamic data. There are some existing researches to investigate the collateral capacity of the CoW, based on 3D model [23,24]. However, these studies just considered two or three general anatomical variations, in order to understand the influences of other variations, more variational cases should also be paid attention. Moreover, according to the recent CTA study of variations of the CoW, the population of China has a higher prevalence of compromised posterior collateral [25]. Therefore, the present work aims to investigate the collateral capacity of the comprehensive CoW with anatomical variations in the posterior circulation.

## Materials and methods

### Basic model reconstruction

**Figure 1:**
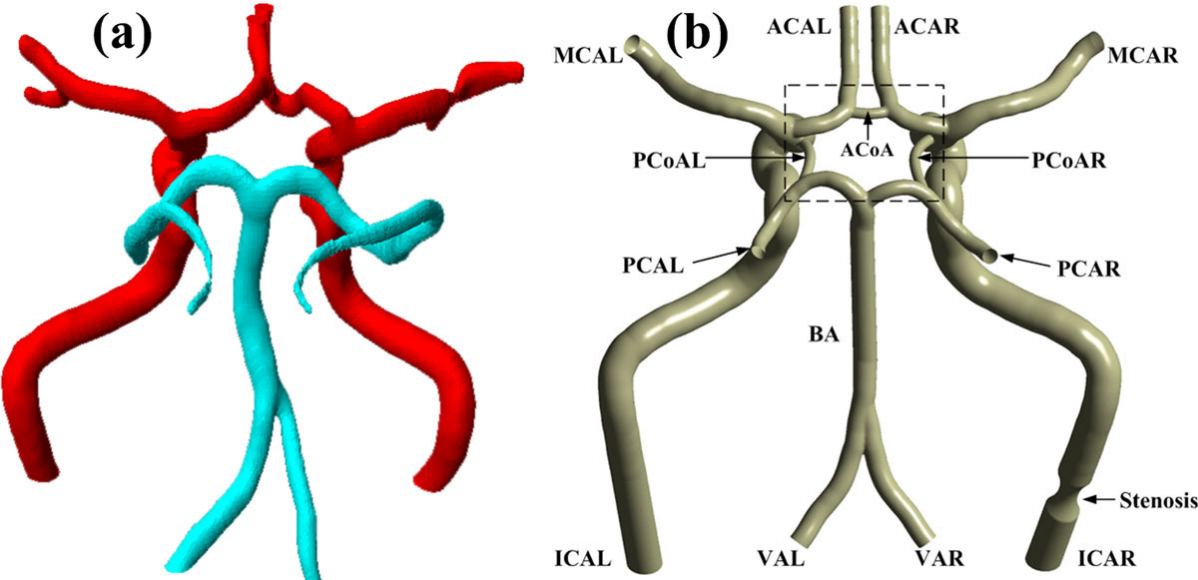
**3D models of the CoW**. (a) Patient-specific model. (b) Ideal model. The abbreviation of each artery branch is marked nearby. The last letter "L" and "R" in the abbreviations represent the left and right side of the arteries, respectively. The black dashed box shows the center of the CoW.

Blood of the CoW is supplied by two internal carotid arteries (ICAs) and two vertebral arteries (VAs), and they are classified as the afferent arteries. Each ICA bifurcates to form one middle cerebral artery (MCA) and one anterior cerebral artery (ACA). The two VAs anastomose to form the basilar artery (BA) and then bifurcate to form bilateral posterior cerebral arteries (PCAs). Wherein, bilateral ICAs and PCAs are connected with left and right posterior communicating artery (PCoAs), while bilateral ACAs are connected with anterior communicating artery (ACoA). The ACAs, MCAs and PCAs are classified as the efferent arteries and transport blood away from the CoW to supply the whole cerebral tissue. Figure 1 shows the 3D geometric models of the CoW with the left denoting a patient-specific model of a normal subject and the right representing an ideal model. The patient-specific model was reconstructed with SIMPLEWARE software combined with MRA data. To obtain the spatial feature of the CoW, we extracted the central line from the patient-specific model. With the help of CAD software (ANSYS, Geometry), the ideal CoW model was reconstructed with each vessel branch assigned a constant diameter which was statistized in Ref. [23] basing on a huge amount of MR data.

**Figure 2:**
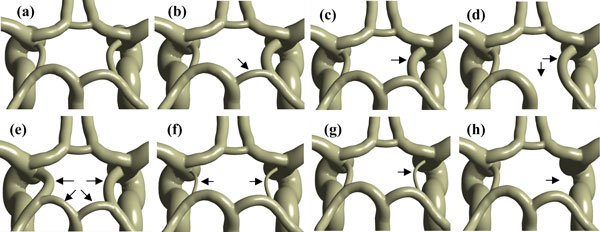
**Anatomical variations of the CoW**. The black arrows point out the variant arteries compared with the normal CoW model. (a) normal-type circle or NT; (b) fetal-type posterior or FTP; (c) transitional-type or TransT; (d) full FTP or fuFTP; (e) bilateral FTP or bFTP; (f) bilateral PCoA hypoplasia or bhypPCoA; (g) unilateral PCoA hypoplasia or uhypPCoA; (h) unilateral PCoA absent or uabPCoA.

Based on angiography and anatomical data, variations of the posterior circulation often occurs in the proximal PCA (PCA-P1) and bilateral PCoAs. So we just changed the geometries of these two arteries but kept the remaining arteries constant, including the distal PCA (PCA-P2). The variations of corresponding artery mainly represented the changes of the arterial diameter, and the possible changes might be enlargement, shrinkage or absence. Here, an arterial segment is considered to be normal if it is visible and its diameter is larger than 1 mm, otherwise, it is hypoplastic [18]. Each vessel diameter of the normal model is adopted from Moore et al. [23], and some artificial vessel diameters are introduced to simulate the variable models, which are shown in Table 1. Finally, we obtained eight basic models, including the normal CoW model, as shown in Figure 2, which reflects the anatomical variations in the posterior circulation of the CoW. The detailed definitions of the eight basic models are described below:

**Table 1:** Diameters of each artery branch [23]. Diameter1 and Diameter2 represent the shrunken and enlarged diameters of the corresponding vessels, respectively.

Vessel	Diameter(mm)	Diameter1(mm)	Diameter2(mm)
ACA	2.4	-	-
MCA	2.86	-	-
PCA-P1	2.13	1.5	-
PCA-P2	2.13	-	-
ACoA	1.47	-	-
PCoA	1.45	0.7	2.13
BA	3.29	-	-
ICA	4.72	-	-
VA	2.8	-	-

(a) Normal type circle (NT): all of the arterial segments are normal, and PCA-P1 has a larger diameter than the PCoA.

(b) Fetal-type posterior (FTP): the diameter of PCA-P1 is smaller than the unilateral PCoA, and the ICA is the main blood supplier to the PCA.

(c) Transitional-type (TransT): the PCoA and PCA-P1 share an equal diameter.

(d) Full FTP (fuFTP): the PCA-P1 is absent, and the PCA arises from the ICA.

(e) Bilateral FTP (bFTP): the variation of FTP occurs on both the two sides.

(f) Bilateral PCoA hypoplasia (bhypPCoA): both the left and right PCoA are hypoplastic, namely, the diameters of bilateral PCoA are less than 1 mm.

(g) Unilateral PCoA hypoplasia (uhypPCoA): only one side of PCoA is hypoplastic, namely, the diameter of unilateral PCoA is less than 1 mm.

(h) Unilateral PCoA absent (uabPCoA): one side of PCoA is absent.

### Stenosed model reconstruction

There are now two major definitions about the stenosis in the ICA. In the North American Symptomatic Carotid Endarterectomy Trial (NASCET), ICA stenosis was classified angiographically: Degree of stenosis (Std) = (1--[narrowest ICA diameter/diameter normal distal cervical ICA]) × 100% [12]. While basing on the same NASCET data, Moneta *et al*. [26] defined the degree of stenosis in symptomatic patients as the ratio of an internal carotid artery to common carotid artery peak systolic velocity (ICA/CCA PSV). Here, we adopt the first definition of stenosis.

**Figure 3:**
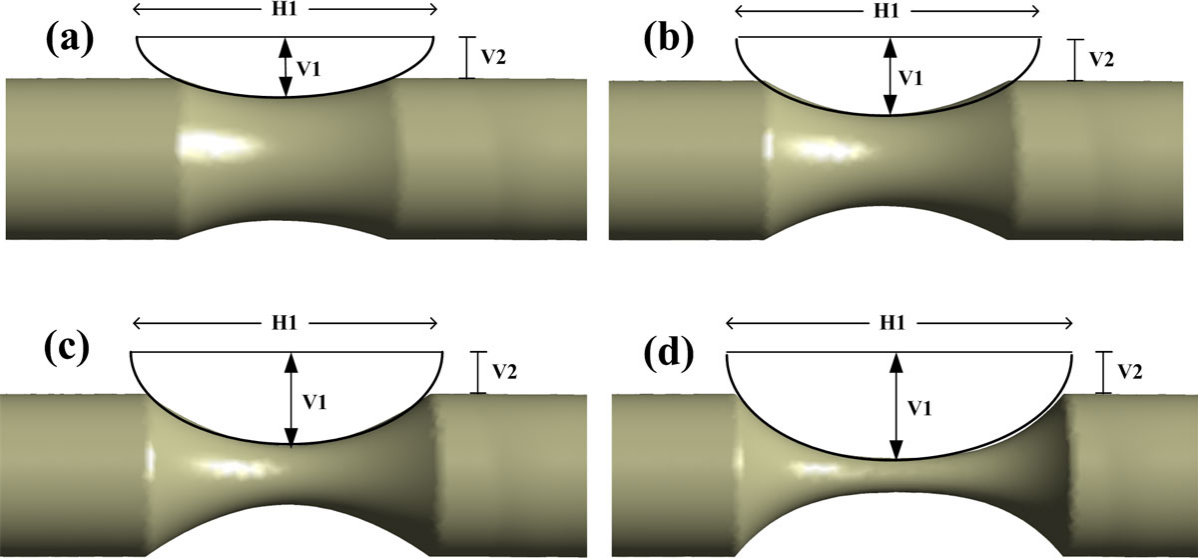
**Schematic diagram of the stenosed models**: (a) stenosis of 24% or Std24; (b) stenosis of 43% or Std43; (c) stenosis of 64% or Std64; (d) stenosis of 79% or Std79. Each semi-elliptical sketch is defined with three parameters: H1, V1 and V2.

In this work, the stenosed site located in the ICAR, which was marked in Figure 1b, and the stenosed models were obtained by cutting the normal vessel in a revolving way with different semi-elliptical sketches, see Figure 3. Figure 3 depicts the four different severities of stenosis based on the first definition above, namely 24% (Std24), 43% (Std43), 64% (Std64), and 79%(Std79), respectively. Table 2 lists the geometric parameters of the four sketches. Besides, the maximum cross-area reduction rates (MCARR) are also listed in Table 2. Eventually, each basic model was derived into four stenosed models and totally 40 models or eight groups with different anatomical variations and different severities of stenosis were studied.

**Table 2:** Dimensions of different sketches and the change of MCARR with different stenosis.

	H1(mm)	V1(mm)	V2(mm)	MCARR(%)
Std24	11	1.7	1.14	42
Std43	11	2.15	1.14	67
Std64	11	2.65	1.14	87
Std79	11	3	1.14	96

### CFD Simulation

Unstructured meshes were created with ICEM CFD (ANSYS, Version 15.0). Element sizes ranged between 0.3 mm and 0.35 mm, with denser elements in high curvature regions. On average, meshes of each model consisted of 0.47 million nodes and 2.5 million elements. Mesh independence were confirmed by performing a denser mesh than those mentioned above. By comparing the VFR difference between different meshes, the relative errors were less than 3%.

Blood was treated as incompressible, viscous Newtonian fluid, and the vessel wall was assumed to be rigid with no-slip boundary condition. Therefore, the governing equations of blood flow are expressed as:

(1) $$\frac{\partial \mu }{\partial x}+\frac{\partial \nu }{\partial y}+\frac{\partial w}{\partial z}=\nabla V=0$$

and the momentum:

(2) ρv.∇v=-∇.τ-∇P

where *P *is the blood pressure, *v *is the velocity vector, *ρ *is the blood density (1056 kg/m^3^) and *τ *represents the shear stress term. The fluid viscosity is set as 0.004Pa.s.

CFD software Fluent 6.3 (ANSYS) was used to perform the simulations. Blood pressure in the realistic physiological conditions is pulsatile, but it's only change the mean cerebral blood flow [23], so we chose the steady pressure as the inlet and outlet boundary condition. As for the choice of pressure, rather than velocity or mass flow, it's because the change of volume flow rate, which reflects the influence of different models, is the indicator to get. Therefore, six outlets were set as a pressure of 75 mmHg, and the four inlets were set as 82.14 mmHg (ICAs) and 80.17 mmHg (VAs). The reason for the different pressure between ICA and VA is that the pressure drops between the two arteries are different. When we choose the real blood flow measured by PC-MRI as the boundary condition [27], the results verify this difference.

In the simulation, the convergence criterion was satisfied when the residual of continuity was less than 10^-4 ^and the velocity component is less than 5.0e-5.

## Results

Here, we only considered the influence of the anatomical variations in the posterior circulation and introduced different severities stenosis in the ICAR, so the other conditions were consistently kept.

### Influence of the degree of stenosis

**Figure 4:**
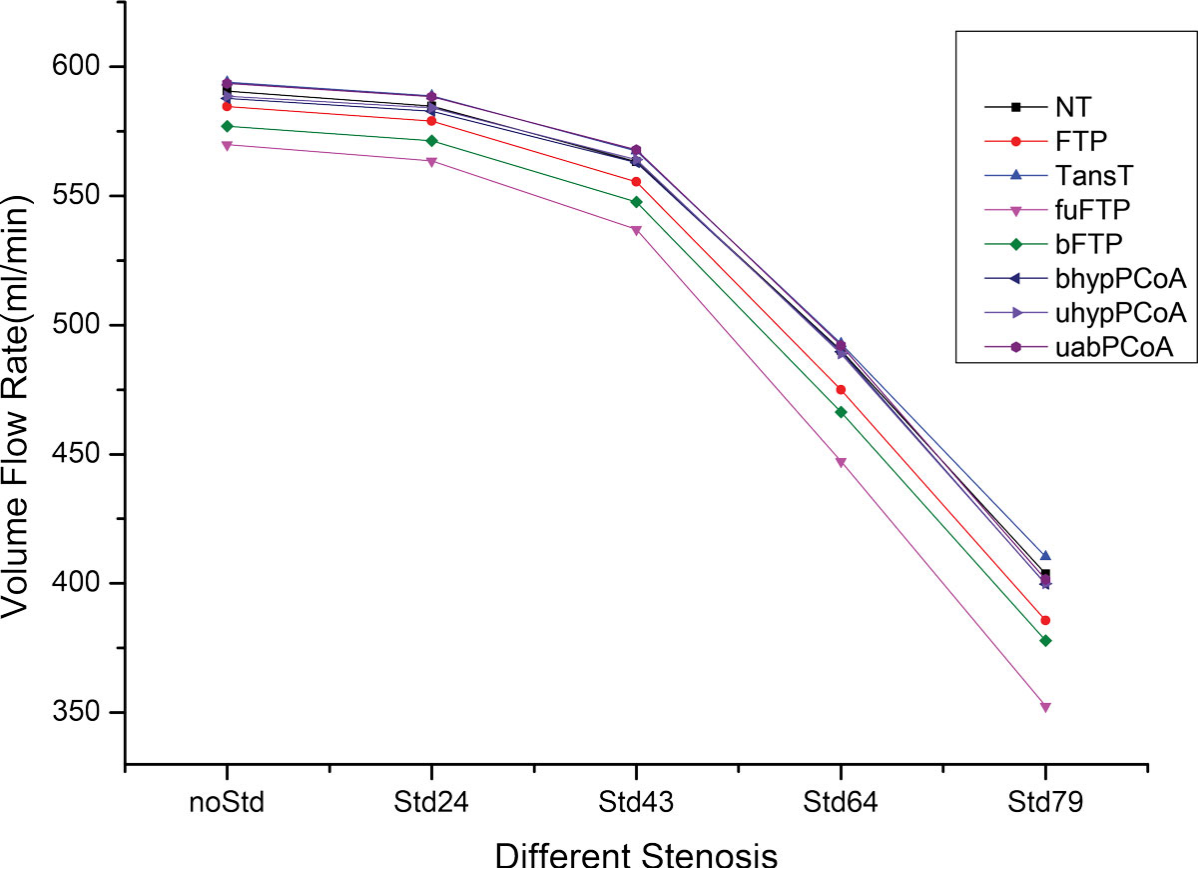
**The VFR change of the inlets for the eight models with different degrees of stenosis located in the ICAR**. (For the meaning of the different colors, please refer to the online version).

Figure 4 presents the variation of total flow rate in the four inlets with degree of stenosis from 0% to 79% for the eight groups of models. According to a present MRA study based on 125 healthy volunteers, the difference of total flow rate between subjects with normal type circle and PCA-P1 hypoplasia circle (fuFTP in this study) is about 18 ml/min (781 ml/min vs 763 ml/min)[28]. Here, in our simulations, we find the corresponding difference is about 20 ml/min (590 ml/min vs 570 ml/min). The difference of total flow rate between our study and the above reference may be that we have neglected some arteries which branch off the ICAs and BAs. In the situation of no-stenosis (noStd), the TransT has the maximum flow rate and the fuFTP has the minimum, and the gap between them is only about 24 ml/min. It is worth mentioning that the anatomical variation of CoW may not result in any symptom in clinic. This is because the cerebral arteries have a series of mechanism to maintain the stable blood perfusion, including collateral circulation and cerebral auto-regulation. With the degree of stenosis increases, all of the models have a decline tendency. Especially, it becomes notable when the ICAR is stenosed over 43%. Therefore, the symptoms of cerebral ischemic syndromes may be notable if the degree of unilateral ICA stenosis is greater than 43%.

**Figure 5:**
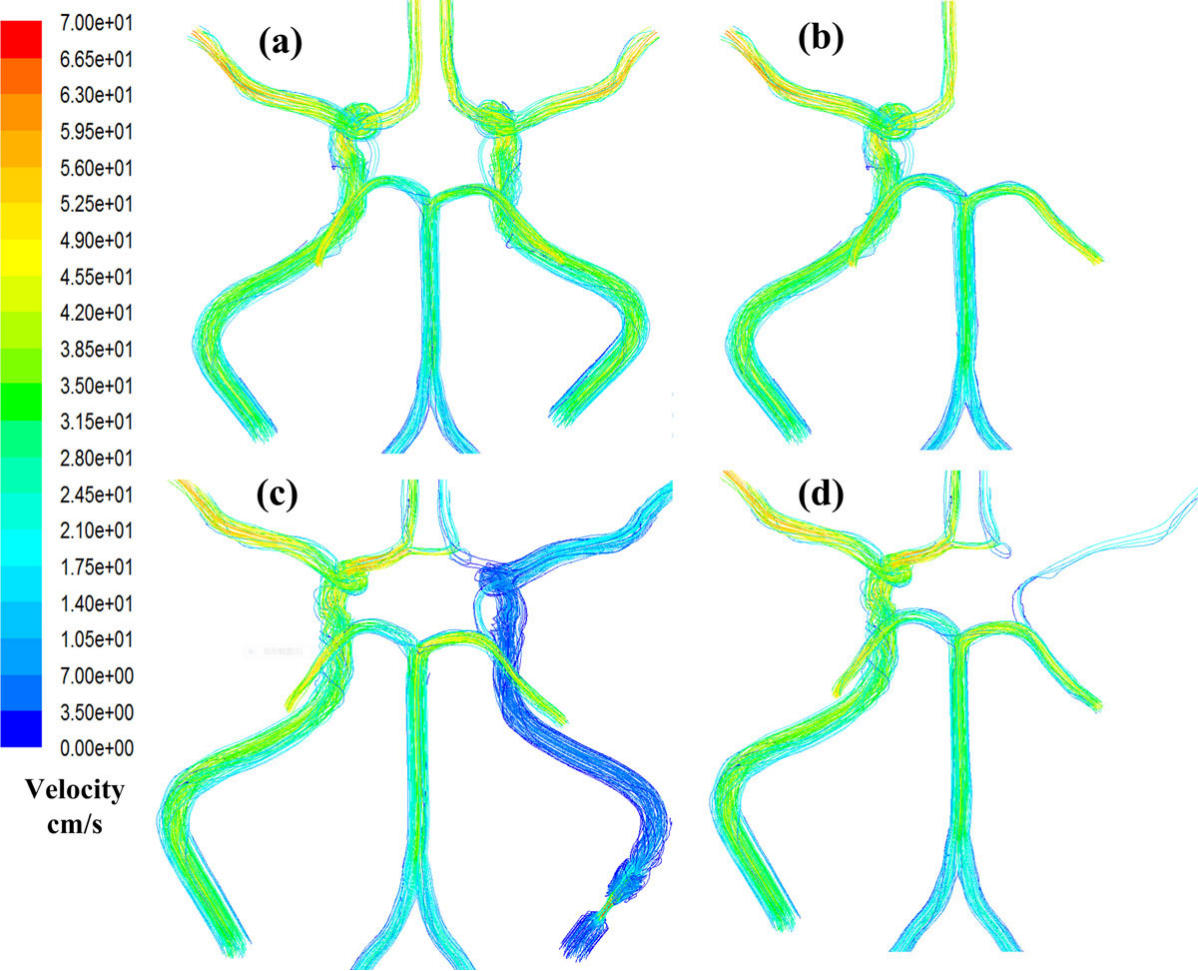
**Streamline distribution of the NT model for the case of noStd and the case of Std79**.

The blood streamline distributions of the NT model with noStd and Std79 are presented in Figure 5. The Figure 5a, b represent the case with no-stenosis of the ICAR, while the Figure 5c, d represent the case with stenosis of 79% in the ICAR. In order to visualize the collateral effect when stenosed, we removed the streamline from the stenosed ICAR and presented the rest streamline (Figure 5b, d). From the figures, we can see that the flow rate distribution is approximately symmetrical for the no-stenosis case. There is almost no flow passing through the ACoA, while the weak blood flow through the PCoAs exists from anterior to posterior. However, for the severe stenosis case (79%), the ipsilateral blood velocity is obviously declined compared with the contralateral blood velocity. In this situation, blood from the ACAL will compensate for the ACAR with the help of the ACoA, and the MCAR will compensated by the PCAR with the help of PCoAR. Meanwhile, we have noticed that there is little blood going through the PCoAL. It can be concluded from the results that the stenosis of ICAR changes the distribution of blood flow and the CoW plays a role in redistributing blood.

### Flow rate variations of MCAs&ACAs with different CoW models

Because the anatomical variations of the CoW are mainly located in the posterior circulation in this study, namely PCA-P1 and PCoA, the PCAs have different flow variations compared with the ACAs and the MCAs. Moreover, the ACAs and the MCAs are the direct branches of the ICAs, so the stenosis in the ICAR will firstly influence the flow rate of the ACAR and MCAR. Based on the above understanding, we will discuss the PCAs from the ACAs and the MCAs separately.

**Figure 6:**
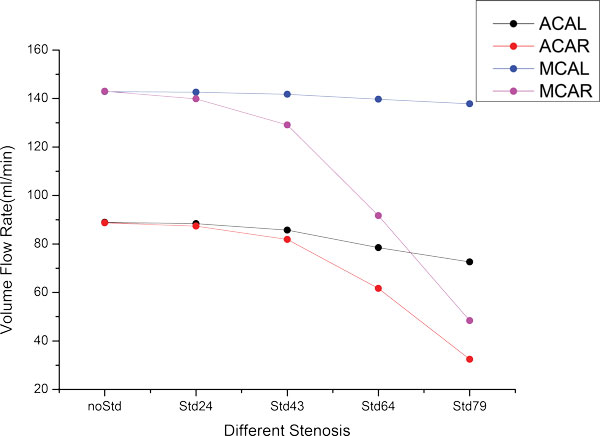
**The VFR change of ACAs and MCAs for the NT model with different degrees of stenosis located in the ICAR**.

Figure 6 shows that degree of stenosis results in the reduction of the VFR in the ACA (Black & Red) and MCA (Blue & Purple) for the NT model. Regarding the total flow rate, its downward trend is very sharp when the degree of stenosis exceeds 43% for the ipsilateral ACA and MCA. In contrast, the contralateral ACA and MCA have a weak decline of the VFR, especially for the MCAL. The remaining 7 models share the same tendency with the NT model.

In order to study the collateral capacity of different models with the severest stenosis (79%), we defined two parameters: ξ_1 _and ξ_2_. If we take the left anterior cerebral artery (ACAL) and the right anterior cerebral artery (ACAR) as an example, the two parameters can be described as below:

(3) $$\xi_{1}=\frac{\text{VFR of ACAL with noStd}-\text{VFR of ACAL with Std}79}{\text{VFR of ACAL with noStd}}*100\%$$

(4) $$\xi_{2}=\frac{\text{VFR of ACAR with noStd}-\text{VFR of ACAR with Std}79}{\text{VFR of ACAL with noStd}-\text{VFR of ACAL with Std}79}$$

where ξ_1 _denotes the percentage of the blood reduction compared with the no-stenosed model, and ξ_2 _represents the relative collateral capacity of ACAR, compared with ACAL. Basing on this definition, other efferent arteries (MCA&PCA) can also be studied in similar way. The two parameters of all considered models are compared in Table 3. From the Table 3, we can see that the larger ξ_1_, the more blood reduction, which means a worse collateral capacity; while the relative collateral capacity of MCAR with respect to MCAL is much greater than that of ACAR with respect to ACAL, which means that the ACAR is better than MCAR in the respect of the relative collateral capacity. This is because the connecting artery of ACAR and ACAL (ACoA) is normal, which ensures blood compensation from the ACAL for the ACAR sufficiently. On the contrary, there is no connecting artery between the MCAL and the MCAR, which results in the high sensitivity of the MCAR to the ipsilateral stenosis. If we compare ξ_1 _of the ACAR and MCAR for each model, the difference of blood reduction rate is not noticeable. However, whether it's for ACAR or MCAR, when comparing ξ_1 _for different models, we find that TransT model has the best collateral capacity, followed by the NT model, FTP model and bFTP model, but the collateral capacity of the fuFTP model and uabPCoA model are the worst. As for the parameter ξ_2_, a smaller value means a better relative collateral capacity and a better connection between the left and right side. We can see from Table 3 that the TransT model has the best relative collateral capacity no matter it's for ACA or MCA. However, when it comes to the worst situation for the relative collateral capacity, ACA occurs in the fuFTP model, but MCA occurs in the uabPCoA model.

**Table 3:** The blood reduction percentage of ACA and MCA compared with their respective no-stenosis model when ICAR stenosed by 79%.

	ξ_1_				ξ_2_	
	**ACAL**	**ACAR**	**MCAL**	**MCAR**	**ACAR/ACAL**	**MCAR/MCAL**
NT	18.3%	63.5%	3.6%	66.2%	3.45	18.55
FTP	17.0%	64.2%	3.4%	66.3%	3.69	18.71
TansT	17.1%	57.6%	3.6%	60.5%	3.32	16.13
fuFTP	16.9%	71.3%	3.1%	72.8%	4.07	21.20
bFTP	18.5%	63.8%	3.2%	66.9%	3.44	21.07
bhypPCoA	18.9%	67.9%	3.4%	70.3%	3.60	20.62
uhypPCoA	19.4%	67.9%	3.5%	70.4%	3.51	20.58
uabPCoA	19.1%	68.8%	3.4%	71.3%	3.63	21.56

### Flow rate variations of PCAs with different CoW models

**Figure 7:**
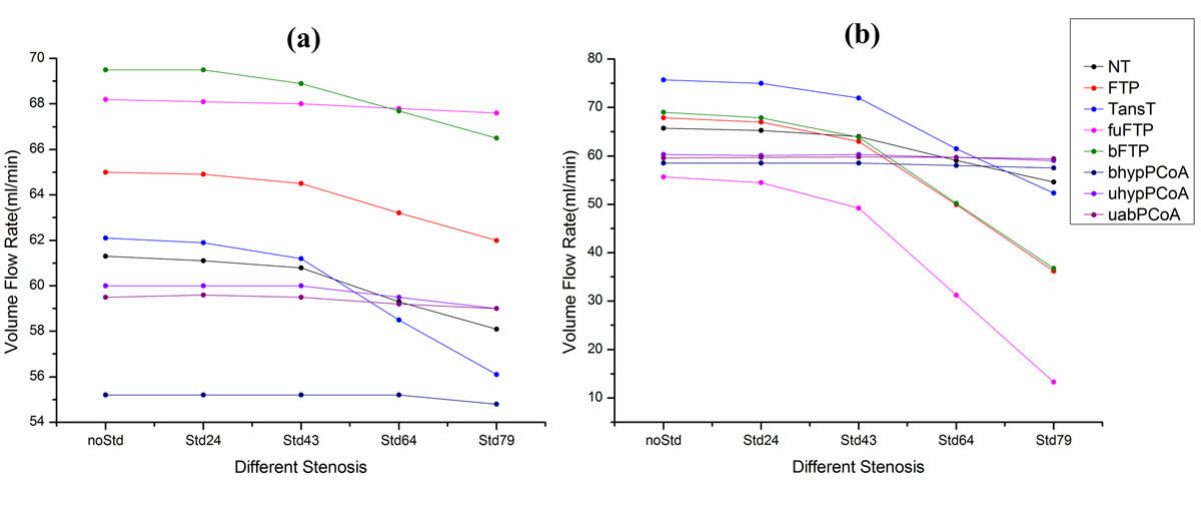
**The VFR change of PCAs for the eight models with different degrees of stenosis located in the ICAR**. (a) Contralateral PCA (PCAL). (b) Ipsilateral PCA (PCAR). (For the meaning of the different colors, please refer to the online version).

Figure 7 presents the changes of VFR of PCAs with different degrees of stenosis for all models. As shown in Figure 7a, flow rate variations of PCAL are not notable with increasing degree of stenosis. For the TransT model, which has the greatest variation, it's only about 6 ml/min of flow rate reduction in the case of 79% stenosis. Nevertheless, the flow rate variations of PCARs present a different characteristic, as shown in Figure 7b. Although the degree of stenosis is enlarging, blood flow of PCARs almost remains the same for the uabPCoA, bhypPCoA and uhypPCoA models. In contrast, the fuFTP model presents a feature of a sharp decline.

In order to quantize the flow rate variations of bilateral PCAs, ξ_1 _is shown in table 4. Comparing the data of different models, we can see that the stenosis (Std79) have the weakest influence on the uabPCoA model, as well as the uhypPCoA and bhypPCoA models. However, it may be fatal to the fuFTP model with 76% blood reduction rate. Meanwhile, as for the cases of bFTP and FTP models, 79% stenosis leads to almost half of blood reduction. Comparing the model geometry, anatomical variations of the PCoAR may play an important role in resulting in these different features. If the diameter of PCoAR is hypoplastic or absent, stenosis in the ICAR can hardly influence the ipsilateral PCA, just as the cases of the uabPCoA, uhypPCoA and bhypPCoA models. On the contrary, PCAR of the fuFTP model branches from the ipsilateral ICA, so the influence is directly and considerable.

**Table 4:** The blood reduction percentage of PCAL and PCAR compared with no-stenosis model when ICAR stenosed by 79%.

	ξ_1_	
	PCAL	PCAR
NT	5.2%	16.9%
FTP	4.6%	46.7%
TansT	9.7%	30.9%
fuFTP	0.9%	76.1%
bFTP	4.3%	46.7%
bhypPCoA	0.7%	1.7%
uhypPCoA	1.7%	2.2%
uabPCoA	0.8%	0.3%

## Discussion and conclusions

The CoW, served as the primary collateral pathway of the cerebral artery, has a major role in maintaining sufficient blood perfusion for brain tissue. A good collateral capacity is beneficial to relieve symptom caused by unilateral artery stenosis or occlusion. The anatomical variations of the CoW lead to the differences for their collateral capacity, and understanding these differences is utmost important for clinicians.

In recent years, the collateral capacity of different configured CoW has been studied. Long *et al*. [24] have studied the collateral capacity of the CoW with severe carotid artery based on 3D models, and this literature mainly concerned about the change of pressure. Alastruey *et al*. [29] have assessed the effects of anatomical variations and occlusions on cerebral flows with 1D computing models, and they have found that ACoA is a more critical collateral pathway than PCoAs if ICA is occluded, and the worst case is that a CoW lacks of the first segment of ACA, and the contralateral ICA is occluded. Fleur *et al*. [30] have studied the influence of FTP on cerebral collateral circulation, and considered that patients with an FTP may be more prone to suffer vascular insufficiency, especially for the case of full FTP.

In this paper, we have reconstructed eight 3D models with anatomical variations in the posterior circulation, and assessed their collateral capacity in term of the flow rate changes. Compared with other studies, the main contribution of this paper is that the anatomical variations in the posterior circulation were investigated and we have considered more variations. The major concern of this study are blood flow rates of various cerebral arteries, however, there is not enough data based on all of the cases in this study, therefore, we just comparing the distribution of flow of afferent and efferent arteries with published PC-MRI measurements [27,28,31] for some special models, such as the NT and fuFTP models. In the case of NT model with no-stenosis, the VFR of unilateral ICA, MCA and ACA with this measurement are about 210-280 ml/min, 130-155 ml/min and 75-90 ml/min, respectively. While in our simulation, the corresponding values are 245 ml/min, 143 ml/min and 89 ml/min, respectively. In the case of fuFTP model with no-stenosis, relative contribution by VFR of the ipsilateral ICA, contralateral ICA and BA is 46.6:41.6:11.7, while we find the specific value is 46.5:43:10.5. The present results agree well with the in vivo data in the literature. In fact, because of the auto-regulation of the cerebral arteries, also including the vast collateral anastomoses and individual difference, it is hard to evaluate the collateral capacity of different configured CoWs quantitatively. However, the tendencies reflected by the anatomical variations are helpful in assessing the collateral capacity of the CoW qualitatively.

In conclusion, with the help of CFD simulation, we have found that the collateral capacities of CoW with anatomical variations in the posterior circulation are different in dealing with unilateral stenosis, and these differences can be reflected by the blood flow rates of efferent arteries. As for the ACAs and MCAs, the TransT model possesses the best collateral capacity. But for the PCAs, unilateral stenosis of ICA has the weakest influence on the uabPCoA model. Meanwhile, we must pay more attention to the fuFTP type for all of the efferent arteries, which have a notable blood flow reduction.

For patients suffering from the ischemic stroke caused by atherosclerosis, it's important to assess the collateral capacity of the cerebral artery, which serves as an important index for selecting the best treatment strategy, especially for the CoW. The study may help neurosurgeons with the risk stratification for patients with cerebral arterial diseases.

Finally, we have to point out the limitations of the work: (1) the results and conclusions are based on ideal models, not the patient-specific models; (2) pulsatile boundary condition has been ignored.

## Competing interests

The authors declare that they have no competing interests.

## Authors' contributions

YR, QC and ZL have made great contributions to the design, analysis and interpretation; YR has been involved in performing the finite element analysis and drafting the manuscript, and QC is responsible for revising it critically; ZL has given final approval of the version and agree to be accountable for all aspects of the work. All authors read and approved the final manuscript.

## Authors' information

YR: M.E., Biomechanics Laboratory, School of Biological Science and Medical Engineering, Southeast University, Nanjing, China; QC: Ph.D, Associate Professor, Biomechanics Laboratory, School of Biological Science and Medical Engineering, Southeast University, Nanjing, China; ZL: Ph.D, Full Professor, Principal Investigator, Biomechanics Laboratory, School of Biological Science and Medical Engineering, Southeast University, Nanjing, China;

## Declarations

Publication of this article was paid with funding from the National Natural Science Foundation of China (NSFC, No 11272091).

This article has been published as part of *BioMedical Engineering OnLine *Volume 14 Supplement 1, 2015: Cardiovascular Disease and Vulnerable Plaque Biomechanics. The full contents of the supplement are available online at http://www.biomedical-engineering-online.com/supplements/14/S1
